# Prognostic role of *BRCA1* mutation in patients with triple-negative breast cancer

**DOI:** 10.3892/ol.2013.1684

**Published:** 2013-11-14

**Authors:** JELENA MAKSIMENKO, ARVIDS IRMEJS, MIKI NAKAZAWA-MIKLASEVICA, INGA MELBARDE-GORKUSA, GENADIJS TROFIMOVICS, JANIS GARDOVSKIS, EDVINS MIKLASEVICS

**Affiliations:** Oncology Institute, Riga Stradins University, Riga, LV-1012, Latvia

**Keywords:** triple-negative breast cancer, breast cancer, *BRCA1* mutation

## Abstract

Triple-negative breast cancer (TNBC) is proposed to be an immunohistochemical surrogate of the basal-like breast cancer subtype. In spite of the relative chemosensitivity of this cancer subtype, it is characterized by aggressive clinical behavior; therefore, a further subclassification of TNBC is required to develop new targeted treatment. In previous studies, a strong correlation between *BRCA1* mutation-associated tumors and TNBC has been identified. The aim of the present study was to investigate the prognostic significance of carrying two germline *BRCA1* founder mutations (4153delA and 5382insC) in patients with TNBC in the Latvian population. A total of 78 consecutive *BRCA1* mutation-negative and 38 *BRCA1* mutation-positive invasive TNBC patients in stage I–IV with no history of ovarian or other primary advanced cancers, who had undergone definitive surgery and genetic testing between 2005 and 2011, were deemed eligible for study. Relapse rates and breast cancer-specific survival (BCS) outcomes were compared between mutation carriers and non-carriers. Univariate and multivariate analyses Cox proportional-hazards models were used to compute independent predictors of survival outcomes. No statistically significant differences were identified in relation to tumor size, T stage, stage, Ki-67 status and tumor differentiation grade between the two groups. The median follow-up period was 36 months for mutation carriers and 41 months for non-carriers. A higher proportion of *BRCA1* mutation non-carriers experienced distant recurrence compared with that of mutation carriers (P<0.03). *BRCA1* mutation carriers had a significantly higher BCS than non-carriers (94.9 vs. 76.9%; P<0.02). In the univariate analyses, *BRCA1*-positive status was associated with decreased risk of distant recurrence (HR, 0.228; 95% Cl, 0.052–0.997; P<0.049) and breast cancer-specific mortality (HR, 0.209; 95% Cl, 0.048–0.902; P<0.036). In the multivariate analysis Cox proportional-hazards model, *BRCA1*-positive status was an independent favorable prognostic factor for distant recurrence-free survival (HR, 3.301; 95% Cl, 1.102–9.893; P<0.033). In conclusion, results of the present study demonstrate that positive *BRCA1* founder mutation status in TNBC, with no evidence of ovarian or other cancer type in advanced stage, significantly improves prognosis.

## Introduction

Triple-negative breast cancer (TNBC) is a heterogeneous clinicopathological entity defined as an estrogen receptor (ER)-, progesterone receptor (PR)- and HER2/neu-negative breast cancer (1). TNBC has been proposed to be an immunohistochemistry (IHC)-based surrogate of the basal-like breast cancer subtype; however, there is no complete overlap between the two groups (2). TNBC accounts for 10–20% of all breast cancer subtypes (2,3). As TNBC is hormone receptor- and HER2/neu-negative, there is no targeted treatment available for this cancer subtype, and a standard chemotherapy regimen remains a basic systemic treatment option, with no optimal cytotoxic regimen recommended. In spite of the relative chemosensitivity of this cancer subtype, it is characterized by aggressive clinical behavior with a high recurrence and mortality rate, particularly in the first 5 years following diagnosis (4). A further subclassification of TNBC is thus required to develop a new targeted treatment to improve prognosis in this unfavorable cancer subtype. In previous studies, a strong correlation between *BRCA1* mutation-associated tumors and TNBC has been identified; 57–88% of all *BRCA1*-associated tumors are TNBC and/or basal-like (5,6). The prevalence/incidence of germline *BRCA1*/*2* mutations in the TNBC subtype is relatively high, accounting for 10.6–19.5% in consecutive patient groups (7,8). *BRCA1*-mutated tumors carry a dysfunctional DNA double-strand break repair mechanism and, therefore, are considered to be sensitive to platinum-based chemotherapy regimens and inhibitors of the poly(ADP-ribose) polymerase (PARP) (9). Theoretically, these agents may also be a new treatment option for TNBC and, at present, several clinical trials are underway to investigate a therapeutic benefit of DNA-damaging agents and PARP inhibitors in this breast cancer subtype (10,11). Understanding the role of carrying a *BRCA1* mutation may be crucial to guide treatment strategies and conduct clinical trials. Therefore, several previous studies have focused their attention on the prognostic role of positive BRCA mutation status in the TNBC subtype and have demonstrated similar outcomes in BRCA mutation carriers and non-carriers (7,12,13). However, these studies are associated with the following limitations: The cut-off levels for ER and PR negativity were not specified or defined as nuclear staining of ≤10% (12,13), neither group was homogenized by received chemotherapy regimens (7), missing information with regard to accompanying cancers or patients with previous ovarian cancer were not included in the study (7,13), breast cancer-specific survival (BCS) rates were not evaluated (7) and the prognostic significance of separate *BRCA1* mutations were also not evaluated (7,12,13). *BRCA1* germline mutation variants result in various changes in the structure of the BRCA1 proteins that impact breast or/and ovarian cancer risk and clinical outcomes. For example, a poorer overall survival of breast cancer *BRCA1* 4153delA mutation carriers compared with 5382insC, has been reported (14,15).

Therefore, the aim of the present study was to investigate the prognostic significance of carrying two germline *BRCA1* founder mutations (4153delA and 5382insC) in patients with TNBC in the Latvian population.

## Materials and methods

### Study population

A total of 2,943 patients with invasive breast cancer between 2005 and 2011 (~50% of all breast cancer cases registered in Latvia during this time period) underwent genetic testing for *BRCA1*/*2* mutations, at the Oncology Institute of Riga Stradins University (Riga, Latvia). In the present study, only patients who met all inclusion and exclusion criteria were included. Inclusion criteria were as follows: i) invasive TNBC in stage I–IV; ii) TNBC defined as ER/PR, 0%; HER2, 0 or 1+ (16); iii) had undergone definitive surgery between 2005 and 2011; iv) tested for *BRCA1*/*2* mutations; v) signed informed consent forms to participate in the study; and vi) had available clinical data. Exclusion criteria were as follows: i) inflammatory breast cancers; ii) a history of ovarian or other advanced cancers; and iii) *BRCA2* mutation carriers. A total of 78 consecutive *BRCA1* mutation-negative TNBCs treated at Pauls Stradins Clinical University Hospital and 38 *BRCA1* mutation-positive TNBCs were deemed eligible for study. The study was approved by the Ethical Committee of Riga Stradins University.

### Pathological examination and IHC

Histological parameters of all cases were reviewed by breast pathologists. Histological type and grade of ductal breast cancers were determined for each case according to the Bloom-Richardson system modified by Elston and Ellis (17).

ER and PR status were determined using IHC. For ER and PR, monoclonal antibodies were obtained from DakoCytomation (Glostrup, Denmark).

HER2 was also assessed through IHC. The assessment of HER-2/neu expression was carried out using the HercepTest kit (Dako, Glostrup, Denmark) according to the manufacturer’s instructions. IHC was scored on a quantitative scale between 0 and 3, in accordance with the Dako HerceptTest™ (Dako).

### Genetic testing

Patients in Latvia were tested for the two common founder mutations in *BRCA1* (4153delA and 5382insC) using a multiplex-specific polymerase chain reaction assay.

### Statistical methods

The outcomes were analyzed in all 116 patients. Locoregional recurrence (LRR) was defined as clinical and histological documented recurrence in the ipsilateral breast, chest wall or regional lymph nodes (axillary, supraclavicular and internal mammary). LRR-free survival (LRFS) was defined as the time from diagnosis to clinical and histological documented evidence of local recurrence. Distant recurrence was defined as clinical and radiographical evidence of distant relapse. Distant recurrence-free survival (DRFS) was defined as the time from diagnosis to first evidence of distant recurrence. The DRFS was censored at the date of the last follow-up if no distant recurrence was observed. The BCS was calculated from the date of diagnosis until the patient succumbed due to breast cancer. Routine follow-up was performed every 3–6 months for 3 years, every 6–12 months for 4–5 years and annually thereafter. The median follow-up from the original diagnosis until analysis was 36 months (range, 8–85 months) in the *BRCA1* mutation non-carriers and 41 months (range, 8–86 months) in the *BRCA1* mutation carriers. Clinicopathological characteristics of *BRCA1* mutation carriers and non-carriers were compared using a χ^2^ and Fisher’s exact test. Univariate and multivariate Cox proportional-hazards models were used to compute independent predictors of BCS and DRFS. The following prognostic variables were analyzed: Age, T stage, nodal status, clinical stage, *BRCA1* status, type of surgery performed, radiation and chemotherapy. BCS was estimated using the Kaplan-Meier method and compared by a long-rank test. P≤0.05 was considered to indicate a statistically significant difference. Statistical analysis was performed using the SPSS software, version 16.0 (SPSS, Inc., Chicago, IL, USA).

## Results

### Patient characteristics

Of the 116 TNBC patients, 38 patients (32.8%) were *BRCA1* mutation carriers and 78 patients (67.2%) were *BRCA1* mutation non-carriers.

### Surgery

All patients underwent definitive surgery. The type of chemotherapy and postoperative radiotherapy received were at the discretion of the multidisciplinary treating team. *BRCA1* mutation carriers were significantly younger at diagnosis than non-carriers (median age, 48.8 vs. 54.4 years, respectively; P<0.034). No statistically significant difference was identified in relation to tumor size, T stage, stage, Ki-67 status and tumor differentiation grade between the two groups. Invasive ductal carcinoma was the most common histological type in the two groups, but *BRCA1* mutation non-carriers were more likely to have invasive lobular carcinomas. There was a higher proportion of lymph node-negative patients in the *BRCA1* mutation carriers group (P<0.004), however, there was no difference in performed lymphadenectomy and sentinel node biopsy between the two groups. A higher proportion of *BRCA1* mutation carriers experienced mastectomy (P<0.001). No statistically significant difference was identified between the two groups in terms of received chemotherapy. *BRCA1* mutation non-carriers were more likely to have received radiation therapy (P<0.027; Table I). A total of three patients (3.9%) from the *BRCA1* carrier group and two patients (5.3%) from the *BRCA1* non-carrier group underwent bilateral salpingo-oophorectomy under the age of 50 years. Prophylactic mastectomy was performed in three *BRCA1* mutation carriers (7.7%). Patients with positive *BRCA1* mutation experienced more bilateral breast cancers than non-carriers [6 (15.8%) vs. 2 (2.6%), respectively].

### Estimates of survival outcomes

No statistically significant difference was identified in the LRR rate between *BRCA1* mutation non-carriers and carriers [3 (3.9%) vs. 1 (2.6%), respectively; P=0.8022]. A total of two patients with LRR in the *BRCA1* mutation non-carriers group underwent mastectomy and one patient underwent breast-conserving surgery; in the *BRCA1* mutation group, one patient with LRR in the right axillary lymph nodes underwent breast-conserving surgery. The LRFS was 5.7 months (range, 4–8 months) in the *BRCA1* mutation non-carriers group and 20 months in the *BRCA1* mutation carriers group.

**Figure 1:**
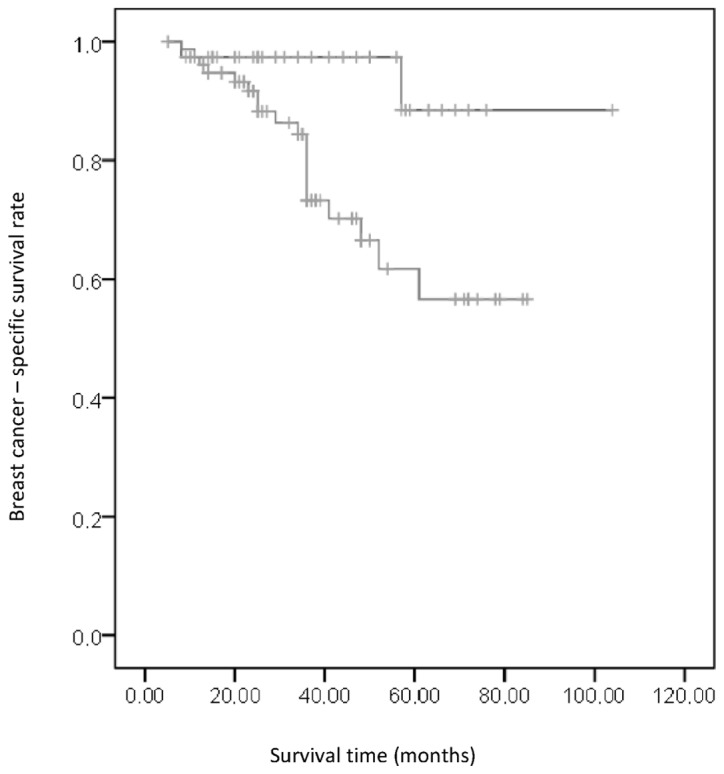
Survival curves of 38 TNBC *BRCA1* mutation carriers (upper line) and TNBC *BRCA1* mutation non-carriers (lower line). P<0.02. TNBC, triple-negative breast cancer.

A higher proportion of *BRCA1* mutation non-carriers experienced distant recurrence compared with mutation carriers [22 (28.2%) vs. 4 (10.5%), respectively; P<0.03]. The DRFS was 32.2 months (range, 6–85 months) in the *BRCA1* mutation non-carriers group and 39 months (range, 9–85 months) in the *BRCA1* mutation carriers group. *BRCA1* mutation non-carriers were more likely to succumb to breast cancer than *BRCA1* mutation carriers [18 (23.1%) vs. 2 (5.3%), respectively; P<0.014]. *BRCA1* mutation carriers had a statistically significant higher BCS than non-carriers (94.9% in the *BRCA1* mutation carriers and 76.9% in the *BRCA1* mutation non-carriers; P<0.02; Fig. 1). The development of bilateral breast cancer did not significantly impact the survival outcomes (HR, 0.040; 95% Cl, 0.001–4.804; P=0.590).

**Figure 2:**
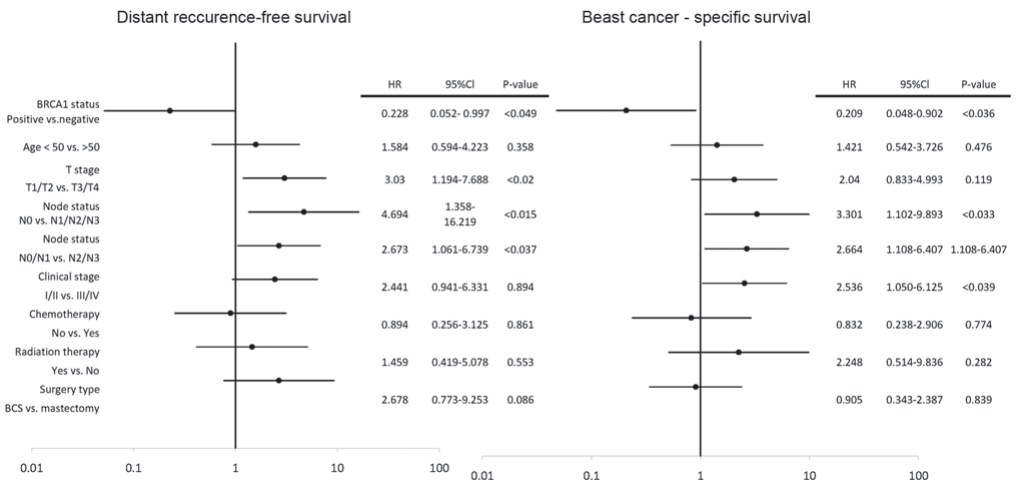
Univariate Cox proportional-hazards model for distant recurrence-free survival and breast cancer-specific survival. HR, hazard ratio; BCT, breast-conserving therapy.

In the univariate analyses, clinical T stage 3 and 4 (HR, 3.030; 95% Cl, 1.194–7.688; P<0.02) and positive lymph node status (HR, 4.694; 95% Cl, 1.358–16.219; P<0.015) were associated with a higher risk of distant recurrence, however, *BRCA1*-positive status (HR, 0.228; 95% Cl, 0.052–0.997; P<0.049) was associated with a decreased risk of distant recurrence (Fig. 2). In the multivariate analyses Cox proportional-hazards model, *BRCA1*-positive status was an independent favorable prognostic factor for DFRS (HR, 0.196; 95% Cl, 1.040–0.965; P<0.045).

**Figure 3:**
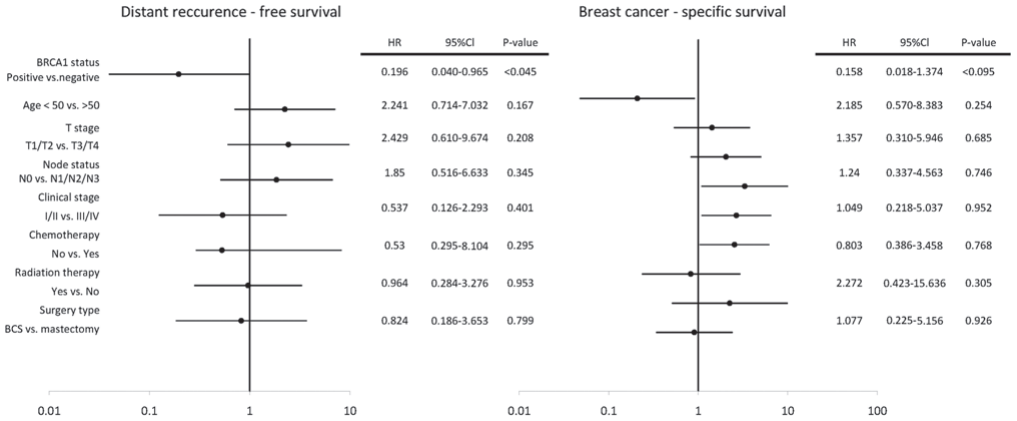
Multivariate Cox proportional-hazards model for distant recurrence-free survival and breast cancer-specific survival. HR, hazard ratio; BCT, breast-conserving therapy.

In the univariate analyses, clinical stages III and IV (HR, 2.536; 95% Cl, 1.050–6.125; P<0.039) and positive lymph node status (HR, 3.301; 95% Cl, 1.102–9.893; P<0.033) were associated with an increased risk of breast cancer-specific mortality, however, *BRCA-1* positive status (HR, 0.209; 95% Cl, 0.048–0.902; P<0.036) was associated with a decreased risk of breast cancer-specific mortality (Fig. 3). In the multivariate analysis Cox proportional-hazards model, no statistically significant effect of evaluated risk factors on BCS was found.

## Discussion

Evidence from the present study indicates that germline *BRCA1* founder mutation (4153delA and 5382insC) carriers, with no evidence of ovarian cancer or other cancers in advanced stage, have significantly improved prognosis, relative to non-carriers. The study demonstrated that positive *BRCA1* mutation status reduces the risk of distant recurrence and breast cancer-specific mortality with statistical significance. Following adjustment for age, T stage, nodal status, stage, surgery, radiation therapy and chemotherapy, positive *BRCA1* mutation status was an independent prognostic factor for lower distant recurrence risk.

Several previous studies have reported no difference or poorer survival outcomes in the *BRCA1* mutation carriers compared with non-carriers (18–20). An equal or improved prognosis for *BRCA1* mutation carriers compared with wild-type was demonstrated; however, this difference was not statistically significant (21). These data were supported by Cortesi *et al*, who identified a statistically significant overall survival advantage in *BRCA1*-positive patients compared with *BRCA1* mutation-negative and sporadic breast cancers (22). None of these studies evaluated the prognostic significance of *BRCA1* mutations in the context of breast cancer subtypes, histological types, tumor grade or received chemotherapy regimens. Several previous studies have focused their attention on the prognostic role of positive *BRCA1* mutation status in the TNBC subtype; however they failed to show a statistically significant improvement in survival for *BRCA1* mutation carriers.

In a study by Lee *et al*(12), the authors reported similar 5-year BCS and overall survival rates in *BRCA1* mutation carriers and non-carriers. In this study, the two groups were well balanced, as all patients received alkylating chemotherapy; however, the definition of TNBC and positivity of ER and PR cut-off levels were not specified. Furthermore, 8% of patients received hormonal treatment.

Gonzalez-Angulo *et al* showed improved recurrence-free survival for *BRCA1* mutation-positive patients treated with surgery and anthracycline-taxane chemotherapy when compared with *BRCA1* mutation non-carriers; however, these patients failed to demonstrate a significant difference in overall survival. The main limitation of this study was that there was a statistically significant difference in received chemotherapy between two groups and, in addition, missing information with regard to other primary cancers and BCS were not evaluated (7). In the study by Bayraktar *et al*, 227 patients with TNBC were included; of 114 BRCA mutation carriers, 94 had a *BRCA1* mutation and 20 had a *BRCA2* mutation. Patients with bilateral and/or metastatic breast cancer and previous breast cancer were not included in the study population. No statistically significant difference was identified in 5-year overall survival rates between *BRCA1*/*2* mutation carriers and non-carriers. Following adjustment for patient age and disease stage, no association with *BRCA1*/*2* mutation status and overall survival was found. In this study, no separate effect of *BRCA1* mutation status on overall prognosis of TNBC was evaluated, negative ER and PR status was defined as nuclear staining of ≤10% and patients with previous ovarian cancer were included in the study (13).

In the present study, a strict criteria of ASCO/CAP guideline recommendations for IHC-based testing of ER and PR was adopted (ER or PR are considered negative if <1% of tumor cell nuclei are immunoreactive) to identify the TNBC phenotype (16), which significantly diminished the number of TNBC cases included in the study. Study data were based on a relatively small number of cases; however, the two groups were homogeneous by tumor grade, the median tumor size, T stage, stage of the disease and received chemotherapy (Table I) and only patients with two common germline founder *BRCA1* mutations (4153delA and 5382insC) were included in the study.

Another difference of the present study was that patients with ovarian cancer and other cancers in advanced stages were not included in the study population. In spite of a significantly improved prognosis for *BRCA1* mutation carriers with ovarian cancer reported by Bolton *et al*, 5-year overall survival for these patients was only 46% (23). In each patient excluded from the study, ovarian cancer was diagnosed in advanced stages (IIIC or IV) and all patients succumbed to disseminated ovarian cancer within a median period of 28.5 months (range, 6–45 months) from the time of diagnosis. The risk of ovarian cancer is ~3% by 40 years old and 54% by 60 years old (24). Several studies have shown a significant heterogeneity of breast and/or ovarian cancer prevalence among various mutations of *BRCA1* gene (14,15,24). Prophylactic salpingo-oophorectomy reduces the penetrance of ovarian/fallopian tube cancer by 75–96% and breast cancer by 56% (25) in patients with the *BRCA1* mutation. In addition, Bayraktar *et al* found that bilateral prophylactic oophorectomy significantly reduces the risk for mortality in patients with TNBC (HR, 0.01; 95% CI, 0.01–0.69; P<0.02) (13).

Improved breast-cancer specific survival in TNBC *BRCA1* mutation carriers compared with non-carriers may be explained by biological differences and/or a higher sensitivity to chemotherapy. In the *BRCA1* carriers group, when compared with the non-carriers group, a higher proportion of node-negative breast cancers were observed (65.8 vs. 37.2%; P<0.004) with no statistically significant difference identified between the T stage of the two groups. A number of studies reported similar data with regard to the prevailing node-negativity in *BRCA1* mutation carriers, even in patients with large tumor size. These may be characterized as one of the main biological features of *BRCA1* carriers. Tumor size and nodal status are independent prognostic factors for survival outcomes. In the univariate analysis, T stage and nodal status, as well as clinical stage, were strong predictors of outcomes. In the multivariate analyses, the factors failed to predict outcomes in *BRCA1* mutation carrier and non-carrier TNBC, perhaps due to a relatively small study population. However, according to Foulkes *et al*, there was no association between increasing tumor size and lymph node positivity in *BRCA1* mutation-positive breast cancers; tumor size and nodal status were weak predictors of outcomes in *BRCA1* mutation carriers (26).

A higher chemosensitivity for *BRCA1* mutation carriers has been proposed in previous studies. Rennert *et al* reported a significantly improved 10-year survival rate for *BRCA1* mutation carriers when compared with non-carriers in patients treated with chemotherapy and no difference in survival rates among patients who did not receive chemotherapy (18). Results of the present study were similar with 89.5% of patients in the *BRCA1* mutation group and 85.9% of patients in the *BRCA1* mutation non-carriers group who received chemotherapy. The heterogeneity of the TNBC phenotype may explain the phenomenon that, regardless of high chemosensitivity, TNBC showed poorer survival outcomes compared with other cancer subtypes.

TNBC is an extremely heterogeneous clinicopathological entity with various prognostic implications and clinical features for pathological and molecular subgroups. The majority of TNBCs are presented by ductal carcinomas (27); however, several other histological breast cancer types may also lack expression of ER/PR and HER2/neu IHC-based staining (medullary, apocrine, pleomorphic lobular, metaplastic and adenoid cystic carcinomas). Apocrine, adenoid cystic and classical medullary carcinomas are associated with favorable prognosis. By contrast, metaplastic TNBC displayed a similarly poor prognosis as high grade adenocarcinomas, but was less sensitive to conventional chemotherapy (28–31). According to gene expression profile studies, TNBC may be divided into several distinct subgroups: Basal-like breast cancer (40–80%), normal-like, claudin-low, interferon-rich, molecular apocrine and HER2-enriched TNBC (32). However, this subclassification of TNBC requires further investigation. A significantly poorer prognosis has been reported for basal-like TNBC when compared with non-basal-like breast cancers. There is an overlap between *BRCA1*-associated cancers, TNBC and basal-like breast cancer. *BRCA1*-mutated tumor cells have a defective homologous-recombination repair pathway that predisposes a high sensitivity to DNA-damaging agents (10). Sporadic TNBC or basal-like breast cancers may also have a dysfunctional *BRCA1* pathway that is caused by epigenetic mechanisms, for example, upregulation of inhibitor of DNA binding 4 (33) or *BRCA1* promoter hypermethylation (34). In studies on an experimental cell system, *BRCA1*-defective cell lines demonstrated higher sensitivity to platinum agents compared with *BRCA1*-competent cell lines and resistance to taxanes (35). Therefore, several clinical trials are currently underway to investigate the role of DNA-damaging agents (platinum-based regimens) and PARP-inhibitors in the treatment of *BRCA1*-associated TNBC (36,37).

In conclusion, the present study demonstrates that positive *BRCA1* founder mutation status in TNBC significantly improves prognosis and may be useful for counseling patients with regard to life expectancy, affecting the choice of chemotherapy regimens and providing the potential for treatment with molecular-targeted therapy. Results of the present study indicate that *BRCA1*-associated TNBC should be considered as a biologically and prognostically distinct subtype of TNBC that displays higher sensitivity to chemotherapy.

## Figures and Tables

**Table I:** Clinicopathlogical characteristics of *BRCA1* mutation carriers (n=38) and non-carriers (n= 78).

Characteristics	*BRCA1* mutation carriers, n (%)	*BRCA1* mutation non-carriers, n (%)	P-value
Age at diagnosis, years			<0.034
Median	48.8	54.4	
Range	27–75	31–82	
Histology			
Ductal carcinoma	26 (68.4)	53 (67.9)	0.9584
Lobular carcinoma	0 (0)	12 (15.4)	<0.006
Medullary carcinoma	5 (13.2)	4 (5.1)	0.16
Tumor grade			
Well-differentiated	0 (0)	0 (0)	
Moderately differentiated	7 (26.9)	10 (18.9)	0.4364
Poorly differentiated	19 (73.1)	43 (81.1)	0.6098
Tumor size, mm	36.2	32.9	0.467
T stage			
T1	7 (18.4)	21 (26.9)	0.3283
T2	24 (63.2)	38 (48.7)	0.1503
T3	3 (7.9)	12 (15.4)	0.2772
T4	4 (10.5)	7 (18.4)	0.7810
Nodal status			
N0	25 (65.8)	29 (37.2)	<0.004
N1	5 (13.2)	23 (29.5)	0.1145
N2	5 (13.2)	15 (19.2)	0.2482
N3	3 (7.9)	8 (10.2)	0.8776
Ki-67	59.8	52.2	0.271
Stage			
I	7 (18.4)	15 (19.2)	0.9329
II	21 (55.3)	33 (42.3)	0.1979
III	8 (21)	30 (38.5)	0.0627
IV	1 (2.6)	0 (0)	0.3276
Surgery			
Breast-conserving	6 (15.8)	36 (46.1)	<0.001
Mastectomy	32 (84.2)	42 (53.9)	
Axillary lymphadenectomy			
No	7 (18.4)	13 (16.7)	0.8075
Yes	31 (81.6)	64 (82)	0.9384
Sentinel node biopsy			
No	31 (79.5)	64 (80)	0.4759
Yes	8 (20.5)	14 (17.5)	0.6882
Chemotherapy			
Yes	34 (89.5)	67 (85.9)	0.1954
Anthracycline-based	19 (50)	45 (57.7)	0.4429
CMF	4 (10.6)	6 (7.7)	0.6162
Platine-based	3 (7.9)	3 (3.8)	0.3940
Anthracycline + taxane	6 (15.8)	12 (15.4)	0.9408
Unknown chemotherapy regimen	2 (10.6)	6 (7.7)	0.6741
None	2 (10.6)	6 (7.7)	0.6741
Radiation			
Yes	22 (57.9)	61 (78.2)	<0.027
No	15 (39.5)	10 (2.6)	<0.001
Bilateral breast cancer	6 (15.8)	2 (2.6)	<0.016

## Abbreviations

TNBC: triple-negative breast cancer
ER: estrogen receptor
PR: progesterone receptor
HER2/neu: human epidermal growth factor receptor 2
PARP inhibitors: poly(ADP-ribose)-polymerase inhibitors
IHC: immunohistochemistry
LRR: locoregional recurrence
LRFS: locoregional recurrence free survival
DRFS: distant recurrence-free survival
BCS: breast cancer-specific survival
ASCO/CAP: American Society of Clinical Oncology/College of American Pathologists
*BRCA1*: breast cancer 1
